# Evaluation of traditional and machine learning approaches for modeling volatile fatty acid concentrations in anaerobic digestion of sludge: potential and challenges

**DOI:** 10.1007/s11356-024-33281-2

**Published:** 2024-04-23

**Authors:** Umar Alfa Abubakar, Gul Sanga Lemar, Al-Amin Danladi Bello, Aliyu Ishaq, Aliyu Adamu Dandajeh, Zainab Toyin Jagun, Mohamad Rajab Houmsi

**Affiliations:** 1 Department of Water Resources and Environmental Engineering, Ahmadu Bello University, Zaria, 1045 Kaduna Nigeria; 2 Department of Botany, Faculty of Biology, Kabul University, Kart-E-Char, Kabul, Afghanistan; 3 School of Built Environment Engineering and Computing, Leeds Beckett University City Campus, Leeds, UK; 4 New Era and Development in Civil Engineering Research Group, Scientific Research Center, Al-Ayen University, Thi-Qar, Nasiriyah, 64001 Iraq

**Keywords:** Gaussian function, Surge functions, Anaerobic digestion, Volatile fatty acid, Machine learning algorithms (MLA)

## Abstract

This study evaluates models for predicting volatile fatty acid (VFA) concentrations in sludge processing, ranging from classical statistical methods (Gaussian and Surge) to diverse machine learning algorithms (MLAs) such as Decision Tree, XGBoost, CatBoost, LightGBM, Multiple linear regression (MLR), Support vector regression (SVR), AdaBoost, and GradientBoosting. Anaerobic bio-methane potential tests were carried out using domestic wastewater treatment primary and secondary sludge. The tests were monitored over 40 days for variations in pH and VFA concentrations under different experimental conditions. The data observed was compared to predictions from the Gaussian and Surge models, and the MLAs. Based on correlation analysis using basic statistics and regression, the Gaussian model appears to be a consistent performer, with high *R*^2^ values and low RMSE, favoring precision in forecasting VFA concentrations. The Surge model, on the other hand, albeit having a high *R*^2^, has high prediction errors, especially in dynamic VFA concentration settings. Among the MLAs, Decision Tree and XGBoost excel at predicting complicated patterns, albeit with overfitting issues. This study provides insights underlining the need for context-specific considerations when selecting models for accurate VFA forecasts. Real-time data monitoring and collaborative data sharing are required to improve the reliability of VFA prediction models in AD processes, opening the way for breakthroughs in environmental sustainability and bioprocessing applications.

## Introduction

Sludge from wastewater, whether liquid or solid, is a complex amalgamation of organic wastes with disagreeable smells and variable solid concentrations ranging from 0.25 to 12% (Talaiekhozani 2019). The composition of sludge is usually the basis for the selection of the treatment and management processes (Mohee et al. 2012; Lim et al. 2016). According to Matsimbe et al. (2022), land application of sludge, involving thickening, stabilization, conditioning, and dewatering, is considered an environmentally acceptable and cost-effective disposal method. Alternatively, anaerobic digestion (AD), which involves hydrolysis, acidogenesis, and methanogenesis phases, is the most preferred approach for sewage sludge stabilization (Gahlot et al. 2022) because of the potential for resource and energy recovery. In the acid phase of AD, microorganisms convert the products of the hydrolysis phase into more soluble organic matter, resulting in the generation of short-chain fatty acids which are mostly volatile (Zhang et al. 2016).

Recent research in AD processes has considered the economic and technological viability of organic waste management systems that produce commercially valuable VFAs (Zhang et al. 2020). Additionally, volatile organic compounds (VOCs), including VFAs, have been proposed as early warning signs for the instability of AD processes (Nie et al. 2023). Consequently, various researchers have examined the process of anaerobic digestion of sludge and the subsequent formation of volatile fatty acids (VFAs). Ding et al. (2017) assessed the efficacy of biological hydrolysis pretreatment on municipal secondary sludge digestion. They discovered that acetic acid was the main volatile fatty acid (VFA) produced during the pretreatment process with a corresponding increase in readily available soluble organics. Rubio et al. reported propionic acid buildup when VFAs were monitored as a control measure during the initialization stages of an anaerobic co-digestion process (Rubio et al. 2022). The authors had to intervene by introducing high VFA-tolerant biomass into the system to ensure a stable process.

To get the most out of VFA production in anaerobic digestion processes, mathematical models can help with control strategies, predict the stability of processes, and identify the best operational parameters (Kirchner 2006). The modified Gompertz equation, a popular model for forecasting VFA concentrations, has limitations when it comes to adjusting to different substrates and situations (Momodu and Adepoju 2021). The Monod, first-order, and Contois models are common kinetic models that have been adopted in AD research, with the anaerobic digestion model (ADM) providing a comprehensive and sophisticated system for modeling AD processes (Xie et al. 2016; Baquerizo-Crespo et al. 2021). However, the use of these models can become complex with heavy data requirements and several state variables and constants that need calibration, necessitating the investigation of simpler models customized to specific conditions (Stiglic et al. 2020). Furthermore, prominent models for estimating VFA concentrations, such as the modified Gompertz equation, have limits in responding to varied substrates and changing conditions (Li et al. 2018).

This necessitates the consideration of Gaussian and surge functions (Ghoor 2019; Kushwaha et al. 2022) and machine learning models, which can contribute to the development of a simplified model for process monitoring and prediction. Dynamic parameter optimization with the help of machine learning solves the problems that models like the modified Gompertz equation have when they try to adapt to different substrates and changing conditions (Jimenez et al. 2015). The initial processes in AD, hydrolysis, and acidogenesis are dynamic processes that lend themselves well to representation by machine learning models (Xiang et al. 2024). Machine learning approaches and Gaussian and surge functions can be useful tools to enable dynamically fine-tuned model settings, resulting in more accurate process predictions over a wide range of scenarios (Taye 2023). Recent use of machine learning algorithms like support vector machines (SVM), artificial neural networks (ANN), and ensemble approaches has helped advance the understanding of the complexities of anaerobic digestion and the prediction of the process (Zhang et al. 2019).

However, even though ANN is the most commonly used ML model well known for high predictive accuracy, sometimes the models can become data-driven with a high degree of complexity which often results in low interpretability of model outputs (Shaw et al. 2022). Therefore, other ML models should be evaluated to determine if a balance of accuracy and interpretability can be achieved, which is essential for understanding complex processes like anaerobic digestion (Byliński et al. 2019). Also, in recent years, emerging machine learning models like generalized additive models (GAM) and the INLA model have gained prominence for their notable accuracy and interpretability (Williamson et al. 2022). Additionally, hybrid models, combining classical kinetics with machine learning, may have the capability to realistically represent the complexities of anaerobic digestion (Narayanan et al. 2021). These models, for example, may use the Gaussian and surge functions for steady-state forecasts while employing machine learning to adapt to changing situations, maintaining accuracy in dynamic scenarios (Zhang et al. 2023b). Such models can improve predictive capabilities by taking into account dynamic substrate features and process condition variations (Mobarak et al. 2023) and subsequently lead to substantial advances in the field by providing rigorous insights into acid concentration dynamics in anaerobic digestion systems.

Hybrid models could become significant tools for academics and practitioners, expediting predictions of anaerobic digestion processes. Therefore, the integration of machine learning, the Gaussian, and surge functions into AD process modeling may provide an advancement in the understanding of the intricacies of anaerobic digestion. This paper aims to provide a template for a dynamic model that possesses an adaptive representation of VFA concentration dynamics during anaerobic sludge reduction. This study should bridge the gap between conventional kinetic models and the complicated realities of anaerobic digestion.

## Material and methods

**Fig. 1:**
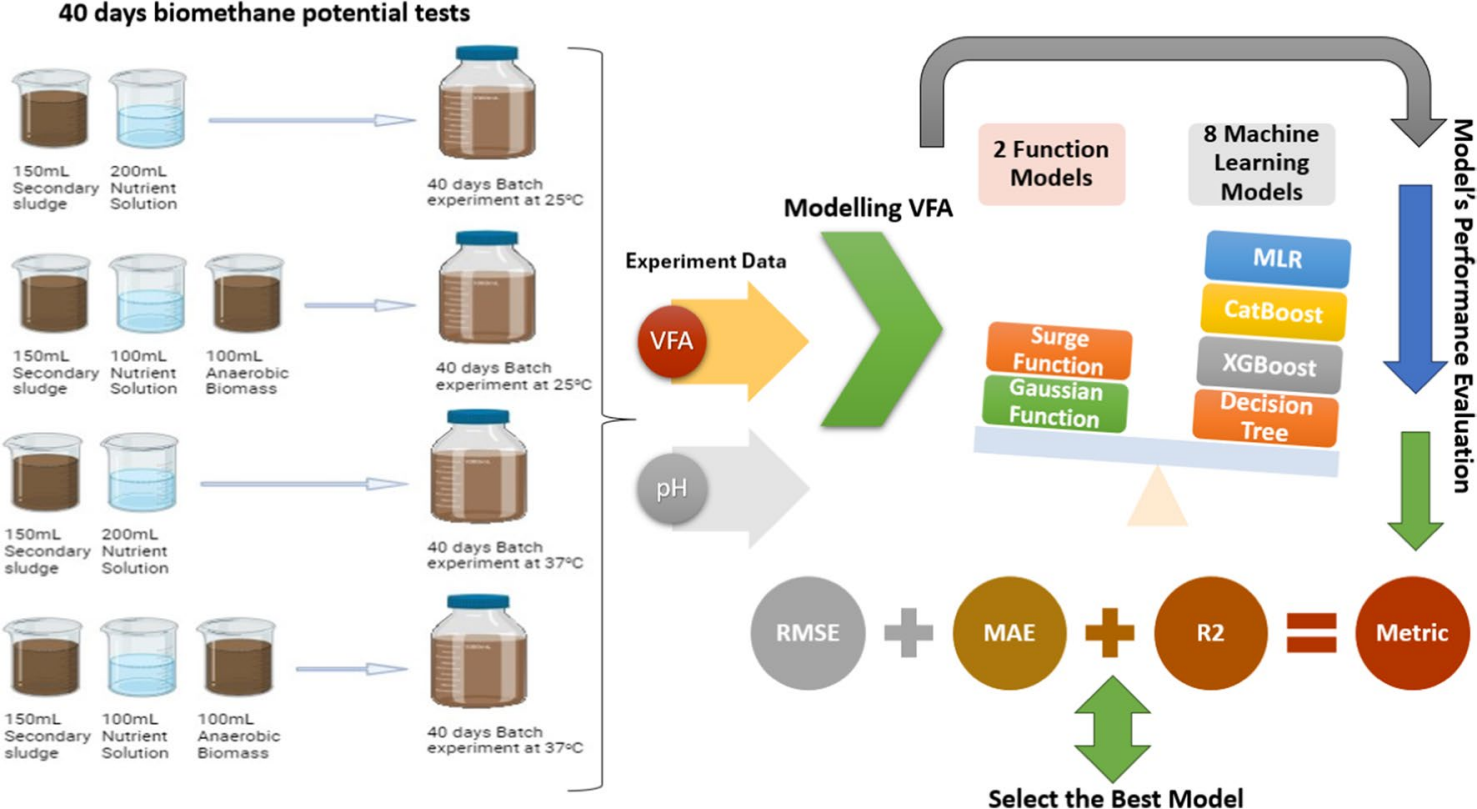
Schematic flowchart of the methodology

This study used anaerobic bio-methane potential tests (Fig. 1) on wastewater treatment sludge, monitoring pH and volatile fatty acid (VFA) concentrations under various settings. Statistical models (Gaussian and Surge) and machine learning methods (Decision Tree, XGBoost, CatBoost, LightGBM, Multiple linear regression (MLR), Support vector regression (SVR), AdaBoost, and GradientBoosting) were used to evaluate the dataset. The best model was selected based on its statistical accuracy using *R*^2^, RMSE, SSE, and MAE as the selection criteria.

### Bio-methane potential (BMP) batch tests

For the bio-methane potential (BMP) batch tests, composite samples (5 L) of primary, and secondary sludge were obtained from various domestic wastewater treatment plants in Scotland through Scottish Water. Anaerobic microbial biomass, obtained from the anaerobic digester at the Hatton wastewater treatment plant in Arbroath, Scotland, underwent a 48-h degassing process at 37 °C. Before initiating the BMP tests, the substrates (primary and secondary sludge) and anaerobic biomass were characterized according to standard procedures, including measurements of initial total solids (TS), volatile solids (VS), pH, and volatile fatty acids (VFA) concentrations. A nutrient solution (NS) comprising specific mineral concentrations in distilled water was introduced to the BMP tests to provide essential micronutrients and trace metals for microorganism growth. The nutrient medium consisted of 75 mg/L ammonium bicarbonate (NH4HCO3), 400 mg/L potassium dihydrogen phosphate (KH2PO4), 5.0 mg/L magnesium sulfate (MgSO4), 5.0 mg/L iron (III) chloride (FeCl3), 5.0 mg/L calcium chloride (CaCl2), 5.0 mg/L potassium chloride (KCl), 1.0 mg/L cobalt (II) chloride (CoCl2), 1.0 mg/L nickel chloride (NiCl2), and 500 mg/L sodium bicarbonate (NaHCO3). The BMP batch tests were conducted in 500-mL glass bottles, each sealed with thick rubber septum and aluminum caps, following recommended anaerobic digestion experiment methodologies (Angelidaki et al. 2009) and prepared in duplicate for each mixture according to the compositions provided in Table 1.

**Table 1:** Three hundred fifty milliliters BMP tests for domestic wastewater sludge

Test ID	Temp. (°C)	Substrate volume (mL)	Anaerobic biomass volume (mL)	Nutrient solution volume (mL)
PS37	37	150 primary sludge	100	100
PS25	25	150 primary sludge	100	100
SS37	37	150 secondary sludge	100	100
SS25	25	150 secondary sludge	100	100
PSnol37	37	150 primary sludge	-	200
PSnol25	25	150 primary sludge	-	200
SSnol37	37	150 secondary sludge	-	200
SSnol25	25	150 secondary sludge	-	200
Blank	37	-	100	250
Blank	25	-	100	250

### Preparation of the BMP batch tests

The BMP batch tests were conducted with meticulous attention to procedural rigor, following established standards for anaerobic digestion experiments (Angelidaki et al. 2009). Glass bottles with a capacity of 500 mL, sealed using a thick rubber septum and aluminum caps, were employed for the experiments, and each experimental condition was replicated. The pH of the final mixtures was carefully adjusted by the addition of a 10 M sodium hydroxide (NaOH) solution. The pH levels were maintained within the range of 7.51 to 7.88. Subsequently, 350 mL of each mixture was precisely measured and dispensed into appropriately labeled bottles (Table 2). To avoid pressure accumulation during methane production, a headspace of 150 mL was left in each bottle. The bottles were securely capped, and the headspace underwent a 2-min flush with pure nitrogen gas, ensuring oxygen-free conditions. Following these meticulous preparations, the bottles were placed in cabinet incubators set at 25 and 37 °C, initiating the commencement of the BMP batch tests.

**Table 2:** Three hundred fifty milliliters BMP tests for domestic wastewater sludge

Test ID	Temp. (°C)	Substrate volume (mL)	Anaerobic biomass volume (mL)	Nutrient solution volume (mL)
PS37	37	150 primary sludge	100	100
PS25	25	150 primary sludge	100	100
SS37	37	150 secondary sludge	100	100
SS25	25	150 secondary sludge	100	100
PSnol37	37	150 primary sludge	-	200
PSnol25	25	150 primary sludge	-	200
SSnol37	37	150 secondary sludge	-	200
SSnol25	25	150 secondary sludge	-	200
Blank	37	-	100	250
Blank	25	-	100	250

### Sample collection from BMP tests

For monitoring of the tests and subsequent parameter analysis, sampling was performed through the septum cap, utilizing Plastipak® 2-mL disposable plastic hypodermic syringes paired with 21-gauge needles (Fisher Scientific, UK). Samples were collected from each test bottle in five 2 mL volumes and then composited to get a 10 mL sample from each bottle. The parameters analyzed were the following:

i. *pH***:** measured using a SensION3 pH probe and meter (Hach Company, Loveland Colorado, U.S.A)
ii. *VFA Concentration:* quantified as acetic acid concentrations (mg/L HOAC) within the range of 27–2800 mg/L and assessed using the ferric hydroxamate method (Hierholtzer et al. 2013), commonly known as the Montgomery method and also Method 8196 as outlined in the DR 5000 user manual. The assessment was carried out using a DR 5000 Hach Lange spectrophotometer (Hach Lange, Salford Manchester, UK). The VFA analysis was performed in triplicates for each sample, with the average of the three measurements serving as the adopted VFA concentration for the respective sample.

### Model description

The approach used for fitting the selected models to the volatile fatty acid (VFA) concentration data obtained from batch tests is based on regression analysis for the Gaussian function and the Surge function. While the machine learning models were developed based on conventional procedures. The models selected for this study were based on the below:

#### Gaussian function

This is a bell-shaped curve commonly used to represent concentration profiles (Eq. 1).

1 $${C}_{VFA}= a* {e}^{\left(-{\left(\frac{t-b}{c}\right)}^{2}\right)}$$

where *C*_*VFA*_ is the concentration of volatile fatty acids (VFA) at time *t*, *a* is the amplitude parameter, representing the maximum concentration of VFA, *e* symbolizes Euler’s natural logarithm constant (2.7183), and *t* denotes the process time, measured in days,* b* is the parameter representing the time at which the maximum concentration of VFA occurs, and *c* is the parameter that influences the width of the curve, affecting the rate at which the concentration of VFA changes over time.

#### Surge function

This incorporates both polynomial and exponential terms, providing flexibility in capturing diverse concentration patterns (Eq. 2).

2 $${C}_{VFA}= a* {t}^{3}*{e}^{-b*t}+c$$

where *C*_*VFA*_ is the concentration of volatile fatty acids (VFA) at time *t*, *e* symbolizes Euler’s natural logarithm constant (2.7183), and *t* denotes the process time, measured in days, *a* is a scaling factor that influences the overall magnitude of the curve, *b* is a parameter that affects the rate at which the exponential term decreases, and *c* is an offset parameter that shifts the entire curve up or down along the *y*-axis.

#### Machine learning models

Unlike the analytical functions, these models do not adhere to a predefined mathematical form (Mowbray et al. 2021); instead, they are developed into complex patterns and relationships by training and testing with available data. Therefore, in this study, supervised learning was adopted for the ML models, and the dataset used for this study consisted of sludge volume, nutrient solution (NS) volume, biomass volume, temperature, time in days, solid mass, and pH as input variables and VFA concentrations as the output variable. ML model development was achieved using the splitting of available data into training, validation, and testing subsets. The ML models were developed and trained on a 70% training dataset, validated on a 10% subset, and tested on a 20% subset (Nikolaou et al. 2021).

### Model fitting and performance evaluation

The fitting of the Gaussian and Surge functions followed a methodology employing the MATLAB curve fitting toolkit (Asadi 2022). For the machine learning model, a suitable algorithm (e.g., regression, neural networks) was chosen, and hyperparameter tuning was performed to optimize its performance (Nematzadeh et al. 2022). In this study, the hyperparameter optimization techniques used were Bayesian optimization, grid search, and random search to enhance model structures (Ali et al. 2023). The performance evaluation metrics used in this study were root mean square error (RMSE), mean absolute error (MAE), error sum of squares (SSE), and coefficient of determination (*R*-squared) as represented in Eqs. 3, 4, 5, and 6.

3 $$RMSE= \sqrt{[ \frac{1}{n} }\sum\nolimits_{i=1}^{n} {(y}_{i}-{\widehat{y}}_{i} ){ }^{2} ]$$

4 $$MAE= \frac{1}{n}\sum\nolimits_{i=1}^{n} {|y}_{i}- {\widehat{y}}_{i}|$$

5 $$SSE= \sum\nolimits_{i=1}^{n} {(y}_{i}- {\widehat{y}}_{i}){ }^{2}$$

6 $$R-squared=1- \frac{\sum_{i=1}^{n} {(y}_{i}- {\widehat{y}}_{i}){ }^{2}}{\sum_{i=1}^{n} {|y}_{i}- {\overline{y} }_{i}|}$$

where *n* is the number of observations, *y*_i_ is the actual value of the dependent variable for observation *i*, *ŷ*_i_ is the predicted value of the dependent variable for observation *i*, and *ȳ*_i_ is the mean of the actual values of the dependent variable.

## Results

### VFA concentrations

**Fig. 2:**
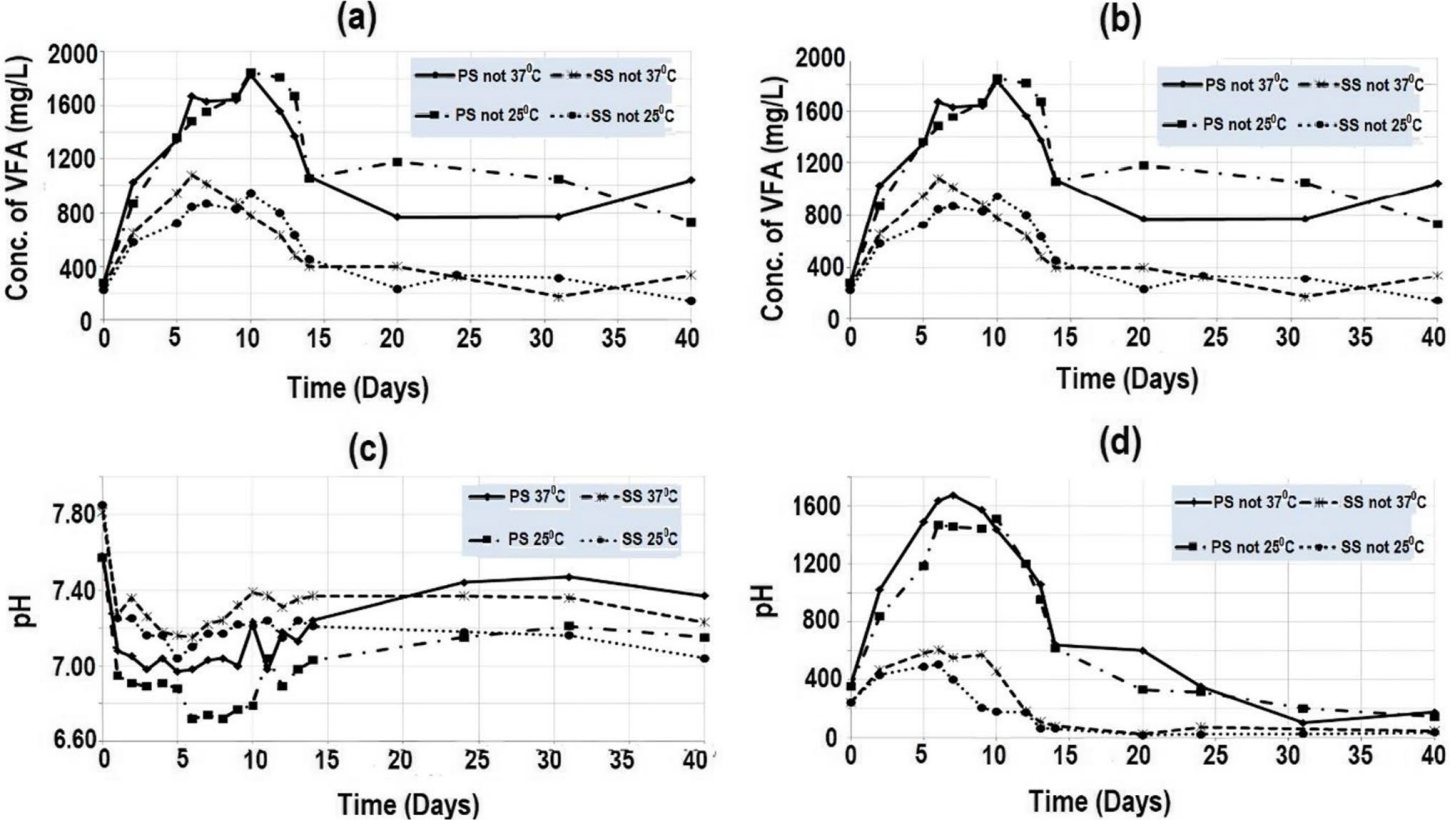
**a** Volatile fatty acids (VFA) conc. (mg/L) from the BMP test of domestic wastewater sludge with anaerobic biomass. **b** VFA (mg/L) from the BMP test of domestic wastewater sludge without anaerobic biomass. **c** Observed pH values from the BMP test of domestic wastewater sludge with anaerobic biomass. **d** Observed pH values from the BMP test of domestic wastewater sludge without anaerobic biomass

This study sought to comprehend and describe the acidogenic phase of AD, which is essential for evaluating the effectiveness of the process (Paranjpe et al. 2023), using BMP tests and modeling of observed data. The BMP investigations showed a significant conversion of primary and secondary sludge into intermediate compounds and changes in the pH of the experiments (Fig. 2).

Figure 2a, b indicates a decline in concentrations of volatile fatty acids in the tests without anaerobic biomass after the first 10 days of the experiment. This decline in VFA concentrations ceased after the 15th day of the experiment even though high concentrations of VFA remained in the tests (greater than 600 mg/L for the PS nol. 25 °C, 300 mg/L for the SS nol. 37 °C, 150 mg/L for the SS nol. 25 °C, and 1000 mg/L for the PS nol. 37 °C after day 40). The decline in VFAs can be attributed to the production of methane, which normally starts after a lag phase of 7–10 days (Rahim et al. 2014). The decline in VFAs over time is consistent with the findings of other studies, where VFA concentration decreased as methane was produced (Rizzioli et al. 2024). Tests with anaerobic biomass (Fig. 2a, b) did not indicate a similar decline in VFAs to tests without biomass; this is likely because microorganisms in the biomass continued to produce VFAs after the initial methane production lag phase (Magdalena et al. 2019).

For the tests with anaerobic biomass, observed VFA concentrations after the 40th day of the experiment were less than 200 mg/L (Fig. 2a, b). The VFA concentrations indicate three stages in the process (Fig. 2a, b), with the first stage being the period during which a continuous increase in intermediate compound concentrations was observed along with a decrease in pH values (Fig. 2c, d), lasting for up to 10 days for all the tests. This stage was followed by the depletion of acid concentrations over a short period of time, between 4 and 8 days (Fig. 2a, b). It shows that the concentration of VFA remains relatively stable during the third stage until the end of the experiment. These stages indicate a curve that is similar to several functions, for example, the Gaussian function, which represents the normal distribution of data in the standard “bell” shape curve, and the surge function, which represents the nature of several natural processes such as the response of human bodies to drug injections. The pH values (Fig. 2c, d) were within the range of 6.0 to 8.0 for all the tests, indicating there was no inhibition of methanogenesis because of pH variation.

### Experimental datasets for fit analysis

The statistical description of the datasets recorded during the 40-day BMP tests is presented in Table 3. The conditions monitored included the sludge volume, nutrient solution and biomass added, temperature, solids mass, pH, and VFA concentrations [mg/L].

**Table 3:** Descriptive statistics of the dataset

Sludge type	Variables	*N*	Minimum	Maximum	Mean	Std. deviation
Primary sludge	Sludge volume (mg/L)	150	150	150	150	0.0
	NS volume (mg/L)		100	200	150	50.35
	Biomass volume (mg/L)		0.0	100	50	50.35
	Temp (°C)		25	37	31	6.04
	Days		0.0	40	11.11	10.43
	Solids mass (mg/L)		9.30	23.98	16.01	3.55
	pH		6.13	7.83	6.86	0.36
	VFA (mg/L)		0.0	1846	1116.79	480.95
Secondary sludge	Sludge volume (mg/L)	150	150	150	150	0.0
	NS volume (mg/L)		100	200	150	50.35
	Biomass volume (mg/L)		0.0	100	50	50.35
	Temp (°C)		25	37	31	6.04
	Days		0.0	40	11.11	10.43
	Solids mass (mg/L)		12.65	25	18.17	3.34
	pH		6.62	7.85	7.08	0.28
	VFA (mg/L)		0.0	1079	462.31	274.10

### VFA concentration fit to the Gaussian function

**Fig. 3:**
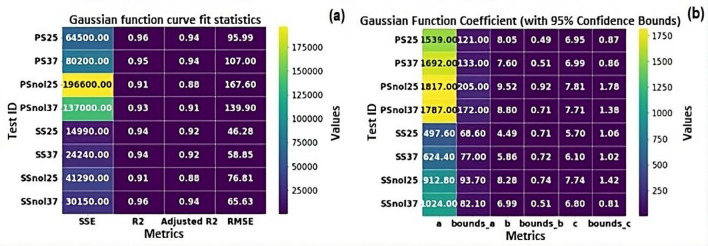
Heatmaps for **a** curve fit statistics and **b** Gaussian function coefficients

The VFA concentration data was subjected to statistical analysis and fitted to Eq. 1, and the correlation was evaluated using RMSE, SSE, and *R*^2^ as metrics (Fig. 3).

The coefficients of determination (*R*^2^) values show if the model can predict the process across time (Palmer and O'Connell 2009). If *R*^2^ is close to 1.0, the function can forecast acid concentrations. If the *R*^2^ value is near 0.0, the function cannot predict acid concentrations or laboratory measurement errors affect the function’s fit to the data. Figure 3a shows that all *R*^2^ values are above 0.90, indicating a good correlation between the Gaussian function and VFA concentration data for all experiments. Figure 3b shows the Gaussian function constants with a 95% confidence range indicating how the Gaussian function coefficients relate to VFA concentration data presented in Fig. 2a, b.

The Gaussian function normally provides a “bell-shaped” probability distribution of a variable, with the coefficients representing process constants. According to Toutiaee and Miller (2020), the Gaussian function only describes the data distribution of a process and does not provide any information on process dynamics. The coefficient “a” in the function represents the possible maximum concentration, which will help manage and design anaerobic digestion processes. Similar observations may be made for the coefficient “b,” which denotes the maximum concentration time. The coefficient “c” indicates the distribution of data points with respect to “b,” the curve’s middle point. Therefore, the Gaussian function mainly provides an assessment of the data in terms of the distribution and does not appear to provide any information in terms of the kinetics of the process; this was similarly reported by Toutiaee and Miller (2020). However, additional studies that explore the extended characteristic of the Gaussian function, such as its fractional derivatives, may be useful for signal processing and control applications (Toutiaee and Miller 2020).

### VFA concentration fit to Surge function

**Fig. 4:**
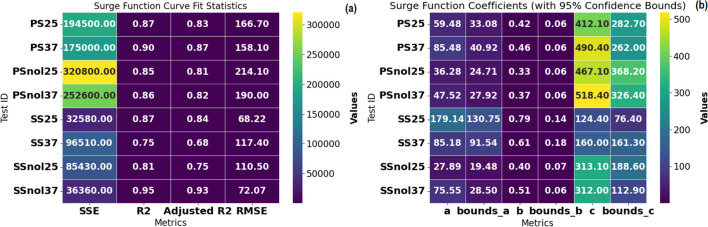
Heatmaps for **a** curve fit statistics and **b** Surge function coefficients

The VFA concentration data was also fitted to Eq. 2, and the correlation was evaluated using RMSE, SSE, and *R*^2^ as metrics (Fig. 4). The *R*^2^ results in Fig. 4a show that the Surge function can accurately forecast volatile acid concentrations for two test conditions: primary sludge with anaerobic biomass at 37 °C and secondary sludge without biomass. However, the *R*^2^ values for the other 6 test scenarios in this study all fell between 0.75 and 0.87, indicating a poor Surge function fit to VFA concentration data (see Fig. 4). This poor fit may indicate data quality rather than model reliability in forecasting VFA concentrations in anaerobic digestion batch experiments. Figure 4b shows the expected Surge function constants with 95% confidence.

From Fig. 4b, coefficient “a” is a system-specific factor, the coefficient “b” is the acid concentration decay or growth rate, and the coefficient “c” is the acid concentration retained at the end of the experiment. It shows that the model predicts minimum values below zero for the SS37 test’s “a” and “c” coefficients within the 95% confidence ranges. All surge function coefficients should be positive (> 0); hence, the surge function cannot accurately describe VFA concentrations during secondary sludge anaerobic digestion at 37 °C. The surge function determines acid concentration by time, which is typical of batch anaerobic systems. When the coefficient “b” is large, then the rate of change is high, and the function will predict a process decay by decreasing acid concentration. The surge function can be useful in determining the ideal digesting period for each system by analyzing residual acid concentration.

### VFA concentration fit with machine learning algorithm analysis

Data preparation is a crucial step in machine learning, as it heavily relies on the specific data within each dataset. To ensure a meaningful comparison across functions and algorithms, the MLA analysis was performed without any data cleansing or additional regularization techniques. This provides a common framework for comparing the findings obtained from the MLA, Gaussian, and surge function fit models.

#### The hyperparameter tuning

**Fig. 5:**
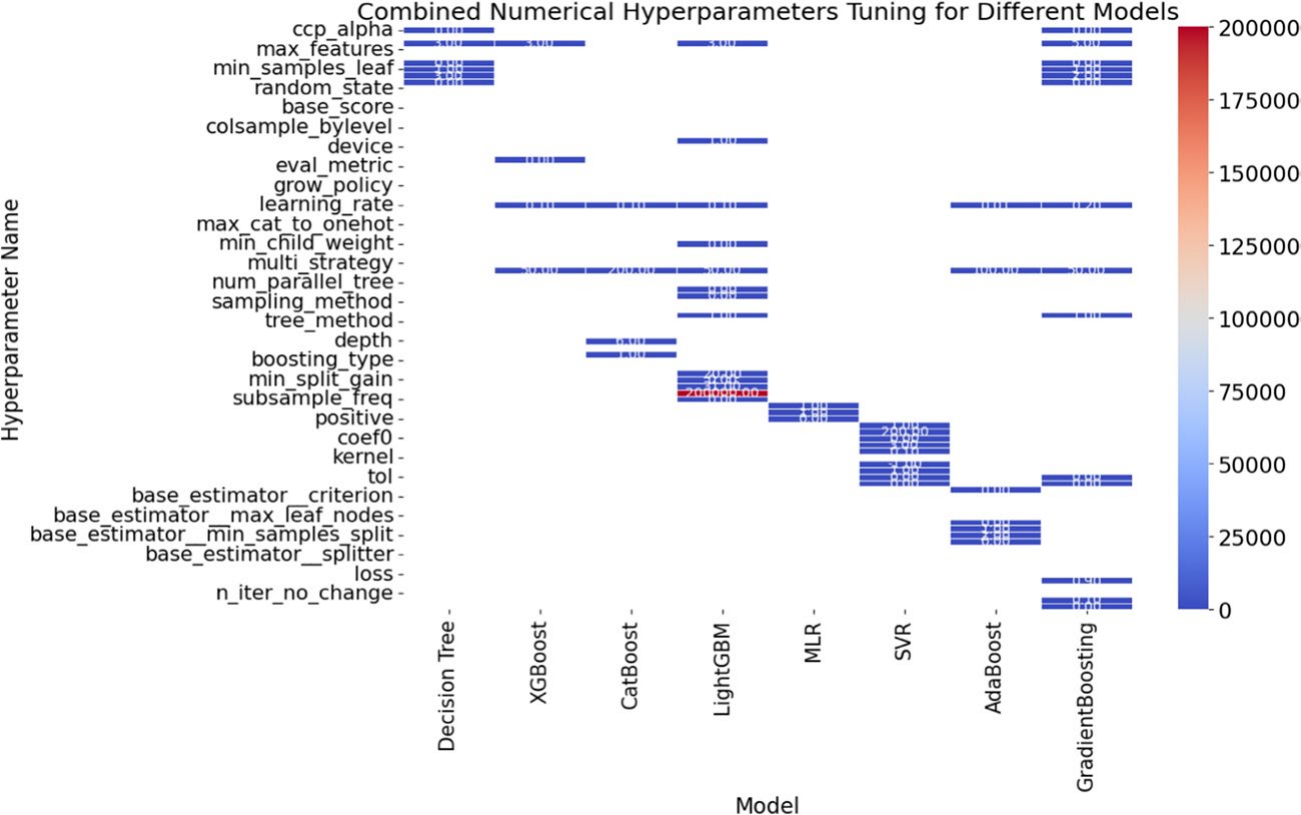
Heatmap of tuning hyperparameter values for the 8 MLA models

The hyperparameter tuning findings from the ML models applied to the primary and secondary sludge dataset provide interesting insights into the models’ complexities and ideal configurations (see Fig. 5). Notably, the decision tree model, which is non-linear, preferred a shallow tree structure with a moderate minimum sample split. This reflects a strategic balance between model complexity and predictive performance, which is consistent with decision trees’ natural interpretability (Florez-Lopez and Ramon-Jeronimo 2015).

For the XGBoost model, which is a robust ensemble approach in ML models, the ideal configuration indicated a small number of estimators, shallow trees, and a moderate learning rate. The model’s emphasis on simplicity and computing economy demonstrates its ability to find a pragmatic balance between complexity and forecast accuracy (Hansen 2020). The CatBoost model, designed to handle categorical variables with ease, preferred a larger number of estimators and a deeper tree structure. This setting highlights the model’s capacity to exploit more extensive data relationships while still balancing the trade-off between complexity and efficiency. Another gradient-boosting approach, the LightGBM model, preferred a small number of estimators, shallow trees, and a moderate learning rate. This mirrors the efficiency and simplicity characteristics that characterize boosting methods. Multiple Linear Regression (MLR), a linear modeling approach, on the other hand, did not require adjustment of standard hyperparameters. Instead, the chosen configuration represents MLR’s fundamental settings, emphasizing its natural simplicity and interpretability. The ideal parameters for the support vector regressor (SVR) with a radial basis function (RBF) kernel comprised a regularization parameter (C) of 1.0 and an epsilon parameter of 0.1. This configuration achieves a careful balance between model smoothness and forecast accuracy. AdaBoost emphasized a larger number of estimators and a lower learning rate by using decision trees as weak learners. This smart hyperparameter configuration option attempts to iteratively reduce mistakes and improve predictive performance (Bischl et al. 2023).

Finally, the gradient boosting model, which used a decision tree ensemble, preferred a moderate number of estimators, deeper trees, and a greater learning rate. This setup indicates the model’s preference for faster convergence and higher predicted accuracy. These hyperparameter combinations expose varied preferences within each model, suggesting a difficult balance between model complications and predictive efficiency (Hutter et al. 2015). These insights are precious for practitioners looking to adjust these models to specific objectives and restrictions in the context of both VFA concentration predictions, ultimately contributing to advances in wastewater treatment procedures.

#### Performance evaluation of machine learning models (MLA)

**Fig. 6:**
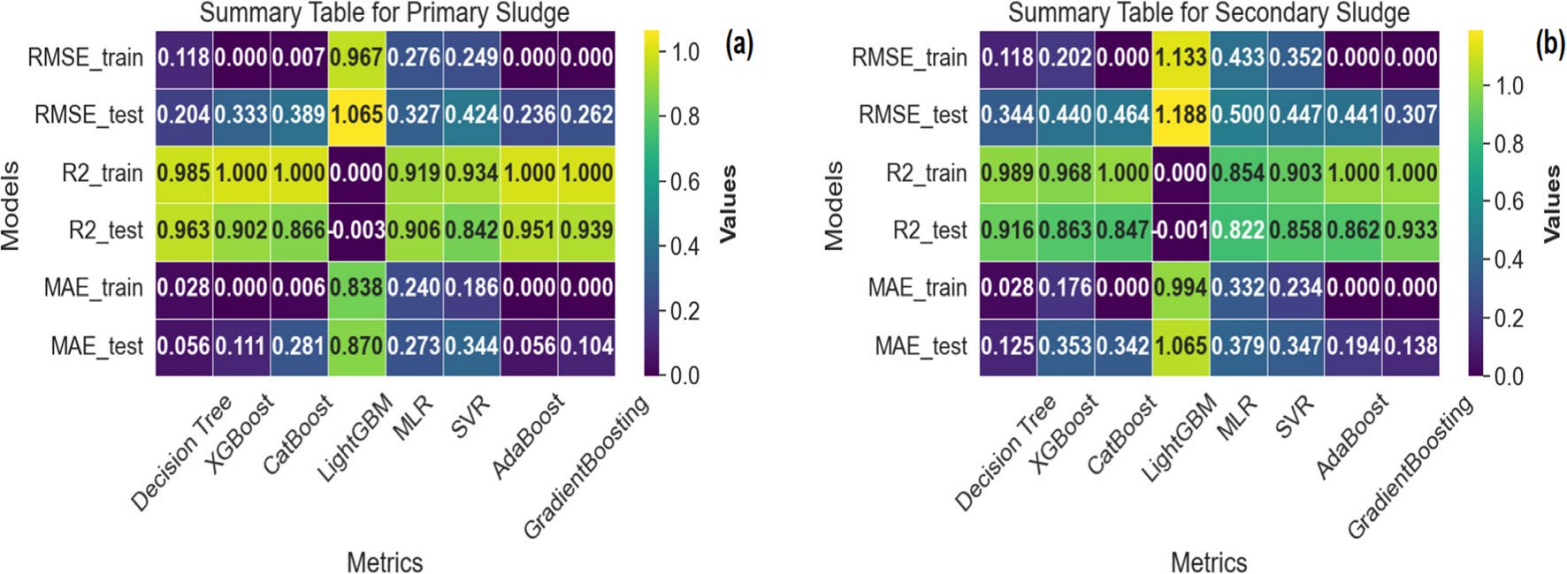
A comparison of the model accuracy test results for the eight MLA models **a** primary and **b** secondary sludge

The performance metrics of multiple machine learning models used to forecast volatile fatty acid (VFA) concentrations in the production process utilizing primary and secondary sludge are presented in Fig. 6. The XGBoost model stands out for primary sludge because it yields particularly low errors, with a training RMSE of 0.000261 and a test RMSE of 0.333350. Furthermore, it achieves an accurate fit with a perfect R-squared value of 1.0 in training and a moderate R^2^ value of 0.902 in testing, exhibiting strong generalization (Fig. 6a, b).

The model’s precision is further emphasized by the low mean absolute error (MAE) numbers. LightGBM, on the other hand, shows a significant disparity between training and testing performance, with an *R*^2^ value near zero in testing, indicating overfitting. Proceeding on to the secondary phase, the Decision Tree model performed well, with an *R*^2^ value of 0.989 in training and 0.916 in testing, demonstrating high predictive skills. While maintaining competitive performance, the XGBoost model has a larger disparity between training and testing *R*^2^ values than the primary method, indicating potential overfitting. Notably, the LightGBM model performs poorly in both types of sludge, with a minimal *R*^2^ in testing. AdaBoost and GradientBoosting consistently produce near-perfect results across both sludge types, demonstrating robust and stable model performance.

#### Selection of the best MLA model

The accuracy test offers a comprehensive examination of eight distinct models utilized for predicting VFA concentrations in sludge processing. The analysis primarily focuses on three performance measures (RMSE, *R*^2^, and MAE). However, choosing the optimal model requires a thorough evaluation that considers factors such as interpretability, computing economy, and forecast accuracy (Hansen 2020). Models such as XGBoost, AdaBoost, and GradientBoosting consistently provide high prediction performance in terms of interpretability. Nevertheless, their collective nature can render them challenging to understand (Demir and Sahin 2023). When conveying findings on the MLA with varying accuracy and capability, model predictability is crucial. In contrast, the Multiple Linear Regression (MLR) model offers a clearer explanation of its coefficients due to its linear nature. The reliability of the model is crucial, particularly when conveying findings on the MLA with varying accuracy and capability (González et al. 2020).

Computational efficiency is another crucial factor that affects the choice of a model. XGBoost, LightGBM, and CatBoost are very efficient algorithms that offer competitive performance while requiring shorter training sessions. Linear models, such as multiple linear regression (MLR), are renowned for their computational efficiency. However, support vector regression (SVR) may entail greater processing demands. Considering both interpretability and computational efficiency, XGBoost emerges as a robust choice for the best model. The consistent and excellent performance of this method on both primary and secondary sludge datasets, along with its ability to balance interpretability and computational efficiency, makes it highly suitable for practical implementation. However, the final choice of model should align with the specific requirements and limitations of the sludge processing application. In cases where interpretability is crucial, MLR can be employed, even if there is a slight decrease in predictive accuracy. Idri et al. (2016) provided that if there are limitations on computational resources, it may be more practical to use simpler models like multiple linear regression (MLR) or ensemble methods like AdaBoost (Idri et al. 2016). For this paper, the best ML model, based on its interpretability, computing efficiency, and the unique requirements of the application, is the XGBoost model, as it demonstrates better model interpretability, computational efficiency, and clearer performance in both primary and secondary sludge when used as VFA sources.

### Performance comparison of the models

**Fig. 7:**
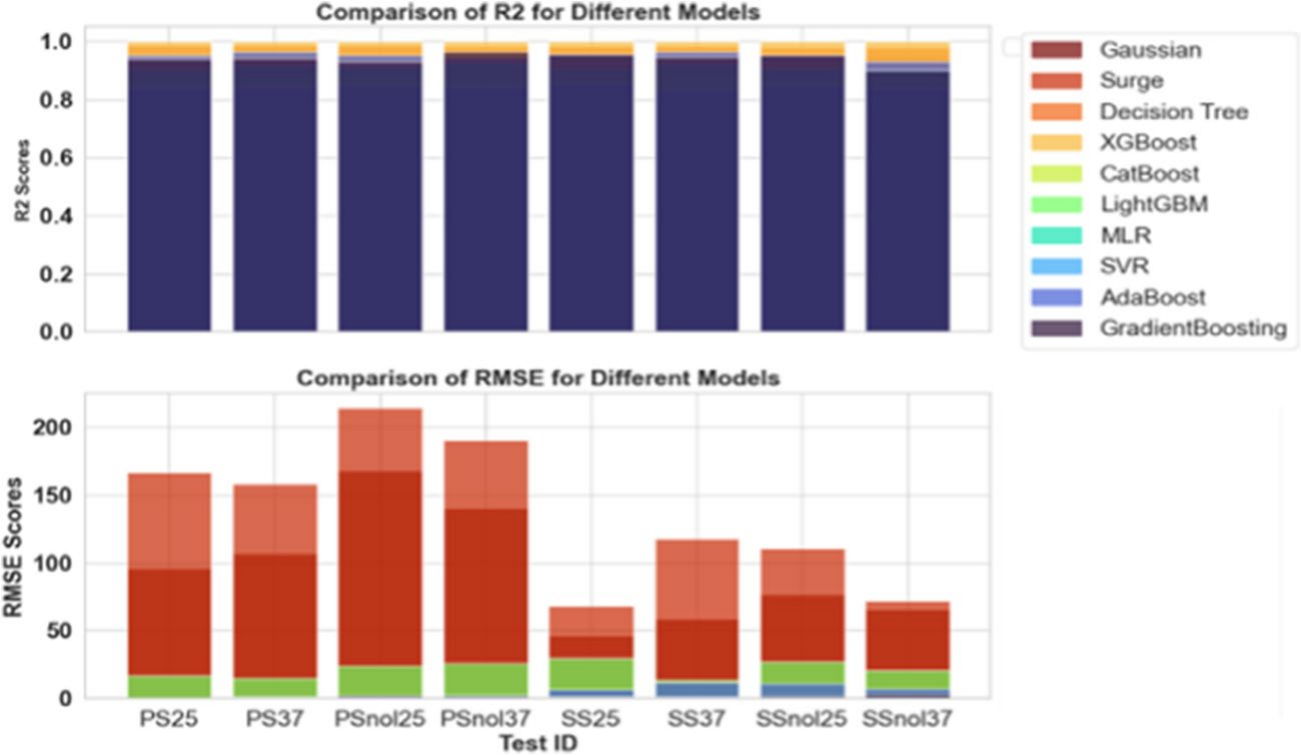
Comparison of the accuracy test of the Gaussian function, Surge function, and machine learning algorithm (MLA) models

When comparing the fit statistics of the Gaussian, surge, and various machine learning algorithm (MLA) models across different test scenarios, both the *R*^2^ and RMSE must be considered (Rahman et al. 2021). These metrics provide information on how well the models capture volatility in the data and how accurate their predictions are. Starting with the Gaussian model, it consistently achieves high *R*^2^ values ranging from 0.8796 to 0.9430 across all test scenarios. According to the *R*^2^ values, the Gaussian model explains a considerable percentage of the variance in the observed data. Furthermore, the matching RMSE values, which range from 46.28 to 167.60, imply rather modest prediction errors. The Gaussian model excels in circumstances such as SS25 and SSnol37, where it gets high *R*^2^ and low RMSE at the same time (see Fig. 7).

The surge model has a wider range of *R*^2^ values ranging from 0.6849 to 0.9312. While the *R*^2^ values are reasonably high, the accompanying RMSE values, which range from 68.22 to 214.10, show larger prediction errors than the Gaussian model. In SSnol37, the surge model performs well, with a high *R*^2^ value; however, the related RMSE is considerably greater. When comparing the MLA models, each has benefits and disadvantages under distinct test settings. The Decision Tree model routinely achieves very high *R*^2^ values (0.979284 to 0.985136) and low RMSE values (0.117851 to 3.242994), indicating that it excels at detecting patterns in data. The XGBoost model is further distinguished by perfect *R*^2^ values (1.0) and low RMSE values (0.33335 to 0.886), indicating a high level of accuracy. Among the MLA models, CatBoost, LightGBM, MLR, SVR, AdaBoost, and GradientBoosting perform differently in different contexts. Notably, AdaBoost obtains high *R*^2^ values (0.9312 to 0.9649) in numerous circumstances but has higher RMSE values in comparison. Gradient boosting has a high *R*^2^ score (0.9012) in SSnol37 but a relatively larger RMSE (Fig. 7).

It is critical to evaluate the unique aims and priorities of the study while choosing between the Gaussian, Surge, and MLA models. If reducing prediction errors is the primary goal, the Gaussian model could be a great contender, especially given its consistent performance across diverse conditions. If attaining the best potential *R*^2^ is a priority, the Decision Tree and XGBoost models outperform the competition. Other MLA models, such as CatBoost, LightGBM, MLR, SVR, AdaBoost, and GradientBoosting, perform differently in different contexts. AdaBoost, for example, achieves high *R*^2^ values but has higher RMSE values. Gradient boosting outperforms in terms of *R*^2^ but has a slightly higher RMSE in SSnol37 (Fig. 7). Finally, the Gaussian model regularly exhibits robust performance with high *R*^2^ and low RMSE across a variety of settings, making it a reliable choice for accurate predictions. While the Decision Tree and XGBoost models have excellent explanatory power, the Gaussian model achieves a good mix of accuracy and simplicity (Zhang et al. 2023a). As a result, the Gaussian model is the ideal choice for this specific research due to its consistent and reliable performance.

## Discussion

The Gaussian model regularly demonstrates high *R*^2^ in many test settings, suggesting its ability to explain a significant portion of the variability in VFA concentrations. The associated RMSE values exhibit a comparatively low magnitude, indicating precise predictions. Gaussian models are renowned in literature for their simplicity and resilience in capturing fundamental patterns in diverse datasets, rendering them well-suited for situations of moderate complexity. Nevertheless, constraints may occur when the fundamental distribution of VFA concentrations greatly diverges from a Gaussian distribution (Zhang et al. 2023a). Although the Surge model has very high *R*^2^ values, it has larger prediction errors, as evidenced by higher RMSE values compared to the Gaussian model. Research indicates that surge models, commonly used in time-series research, can be very responsive to outliers and may have difficulties in accurately capturing nuanced fluctuations in intricate datasets. The fundamental properties of the surge model may impede its efficacy, especially in circumstances with a broader spectrum of VFA concentrations. Researchers must consider the limits of the surge model when dealing with different and dynamic VFA concentration patterns.

Within the many MLA models, the Decision Tree model constantly demonstrates exceptional performance, as evidenced by its notably high *R*^2^ values and low RMSE values. This highlights the model’s strong capability to accurately capture complex patterns in VFA concentrations. Decision Trees are widely recognized in the literature for their interpretability and versatility, which makes them highly important in environmental modeling. Nevertheless, it is important to consider the apprehensions regarding overfitting, particularly in situations involving data that is prone to noise. Furthermore, the intricacy of Decision Trees can restrict their capacity to be applied to unfamiliar datasets. XGBoost, a widely used ensemble approach, has impeccable *R*^2^ values and minimal RMSE values; hence, displaying its exceptional accuracy in forecasting VFA concentrations. The academic literature emphasizes the efficacy of XGBoost in managing non-linear connections and capturing intricate interactions among variables. Nevertheless, the opaque structure of the system presents difficulties in terms of comprehensibility, underscoring the importance for researchers to strike a compromise between predicted precision and model clarity. CatBoost, LightGBM, MLR, SVR, AdaBoost, and GradientBoosting demonstrate diverse performance in different scenarios. AdaBoost achieves high *R*^2^ values, but at the cost of increased RMSE values, highlighting the trade-off between explanatory power and prediction accuracy. GradientBoosting demonstrates superior performance in terms of *R*^2^ in certain situations, while it may result in slightly higher prediction inaccuracies.

Although these models provide useful insights, they nonetheless have inherent limits. Overfitting, a frequent occurrence in machine learning algorithms (MLAs), can undermine the ability of models to generalize to unfamiliar datasets. The performance of the model may be affected by the quality, representation, and biases of the dataset. The presence of non-linear interactions and unpredictable factors presents difficulties, particularly in the field of environmental studies where the dynamics of systems are intricate. Moreover, the interpretability of models, which is essential for decision-making, may be undermined in highly accurate but intricate models such as XGBoost.

Ultimately, the examination of VFA concentrations forecast accuracy from the Gaussian, Surge, and MLA models yields a comprehensive comprehension of their respective advantages and constraints. Researchers must carefully select models based on the study’s goals, considering the compromises between accuracy, interpretability, and generalizability. Continuous progress in improving model accuracy, validation methods, and incorporating specialized expertise is crucial for addressing existing constraints and improving the reliability of VFA prediction models.

## Conclusion

The examination of models for forecasting volatile fatty acid (VFA) concentrations in sludge processing has provided significant insights into their respective strengths and limitations. The Gaussian model consistently demonstrated solid performance, with high *R*^2^ values and low RMSE across a variety of scenarios, making it a reliable choice for minimizing prediction mistakes. However, when VFA concentrations deviate greatly from a Gaussian distribution, extreme caution is advised. While the Surge model had good *R*^2^ values, it had greater prediction errors, indicating possible difficulties in capturing varied changes, especially with a wider range of VFA concentrations. This emphasizes the importance of exercising caution when using the Surge model to varying and dynamic VFA concentration patterns. Among the machine learning algorithm (MLA) models tested, the Decision Tree and XGBoost consistently outperformed others, highlighting their applicability for scenarios requiring the best *R*^2^. Concerns with overfitting and the trade-off between model complexity and generalization, especially given XGBoost’s opaque structure, must be addressed in any future study. AdaBoost and GradientBoosting both displayed distinct strengths, such as high *R*^2^ values, but at the penalty of increasing RMSE, illustrating the trade-off between explanatory power and prediction accuracy. Other models, such as CatBoost, LightGBM, MLR, and SVR, performed inconsistently in various settings, needing careful assessment based on the study’s objectives. Finally, the paper gives a thorough assessment of the strengths and limits of several models in projecting VFA concentrations, paving the way for future advances in environmental sustainability and bioprocessing applications. The study also identified critical gaps and research opportunities for improving the reliability of VFA prediction models, such as rigorous validation on separate datasets, real-time monitoring systems, and collaborative data-sharing activities among research institutions are essential.

## Data Availability

The manuscripts’ data is contained in the text.
